# Scandal mining: political nobodies and remediated visibility

**DOI:** 10.1177/0163443717734408

**Published:** 2017-10-25

**Authors:** Daniel Trottier

**Affiliations:** Erasmus University Rotterdam, The Netherlands

**Keywords:** digital media, online shaming, political scandal, social media, surveillance, visibility

## Abstract

This article considers the 2015 federal election in Canada as the emergence of seemingly citizen-led practices whereby candidates’ past missteps are unearthed and distributed through social and news media channels. On first pass, these resemble citizen-led engagements through digital media for potentially unmappable political goals, given the dispersed and either non-partisan or multi-partisan nature of these engagements. By bringing together journalistic accounts and social media coverage alongside current scholarship on citizenship and visibility, this case study traces the possibility of political accountability and the political weaponisation of mediated visibility through the targeted extraction of candidate details from dispersed profiles, communities and databases.

## Introduction

Electoral strategy is typically directed by major political parties, notably in the collection and analysis of voter information through big data extraction (Bennett, 2013). Recent developments suggest an augmentation of scope and impact of the use of digital tools by a range of unexpected and often unidentifiable actors. Consider Cambridge Analytica, a company claiming to manipulate public sentiment and voting intensions by monitoring and manipulating social media content for specific sub-populations (Madrigal, 2017). Such manipulations occur through big data, which can simply refer to large data sets, or more importantly set of practices surrounding the collection, storage and analysis of large scales of personal information, (Boyd and Crawford, 2011). Likewise in the 2016 American and 2017 French elections, targeted data leaks (Auchard and Felix, 2017; Follis and Fish, 2017) sought to compromise political candidates and shift public opinion. These popularised instances of hacking and dumping require skills and other forms of capital beyond the capacities of most citizens. And while citizens are enrolled through social media platforms in campaigning efforts, in many cases this is limited to sharing and re-tweeting party-approved content.

Yet citizens (among others) have begun to unearth and distribute political candidates’ past missteps through digital and conventional news media channels. This is accomplished by searching through a digital media landscape that includes social media content as well as political and person statements made elsewhere online, including political websites and comment sections on news webpages. Although social media databases are often used in big data analysis, individual users are restricted in terms of their access of this data at any given moment, as well as the types of analysis they can perform. These developments suggest a fledgling electoral sousveillance (Mann et al., 2003), in which those typically under watch are able to render visible the private and discrediting details of political candidates. This also potentially furthers the myth of a big data commons (Berliner Gazette, 2017) that contributes to a kind of new political visibility in which individuals access and utilise data in order to render political parties knowable and accountable. In particular, by publicising objectionable content found online, individuals seemingly unaffiliated with political or media organisations shaped how the 2015 Canadian election is understood, furthering a ‘hybridization’(Chadwick, 2011) of news production.

Revelations of scandal largely depended on journalists’ access to discrediting information about political actors. Access to such information has historically been limited, due to the comparatively lessened visibility of political actor’s private lives, as well as the role of gatekeepers that granted or denied access to less visible aspects of political life. While citizens were largely relegated as receivers of news about political scandal, the current digital media landscape appears to enable them to identify discrediting information about political actors. Scandal mining refers to practices where individuals or organisations actively search targeted political actors on open or otherwise accessible data sources, including but not limited to popular social media platforms. As mediated political interventions, they appear understated in comparison to the events described in the opening paragraph. Yet, in practice, this amounts to more than simply following candidates on social media, but rather a pre-emptive scrutiny of any available data source. The criteria for offence are broad, notably if not limited by the searcher’s own ideological commitments. It is perhaps more accurate to frame scandal mining as a deep data practice (Kendall – in Brock, 2015) as it involves a prolonged and scattered scrutiny of archived and often long-forgotten content. While this may resemble earlier searches in court records and other public documents – including open sources like so-called ‘sunshine lists’ in the Canadian context^1^ – the pervasive nature of platforms like Facebook and Twitter allows for a greater range of details and disclosures. In principle, it is possible to yield evidence of corruption and other abuses of power when searching political candidates’ records (and indeed, this is the case in the context of the 2015 federal election in Canada). Yet the fact that candidates with virtually no political experience are also being pre-emptively targeted means that scandal mining also identifies a broader range of gaffes and missteps, especially when these are sufficient to discredit a political actor.

While scandal mining does not stand entirely under the rubric of big data, these practices may still be made meaningful by assumptions that one can source actionable information about people on social media platforms and other data sources. Media scholarship should consider the manner in which scandal mining is differentiated from earlier manifestations of political scandal and missteps, in particular the role of user-led practices in recent elections. This stands in contrast to the role of news media in earlier political scandals, as well as the role that citizens have formerly occupied in these instances. While news media were the platforms where evidence of scandal and public outrage has been communicated, a broader range of actors through any conceivable online media platform may now take on these roles, notably as offences also occur on these platforms. This article considers the 2015 federal election in Canada as an exploratory case study of scandal mining. During this election, no less than 39 candidates from four major parties were forced to repent or resign as a result of past content made visible through digital media searching. On first pass, this seems like a user-led enrolment of personal content for potentially unmappable political goals, given the dispersed and either non-partisan or multi-partisan nature of these engagements. Several high-profile scandals in the Canadian election were led by self-proclaimed political ‘nobodies’^2^ manually searching available data sources. Yet this election also saw the furthering of scandal mining as party strategy. Alongside supposed political nobodies, veteran political strategists are attempting to assert control over this landscape through specialised and prohibitive software targeting public and private social media content, as well as other open and less open sources including ‘old school visits to court and registry clerks’.^3^

The emergence of scandal mining partly stands as a distributed and potentially democratic relationship between public and data, but one that is arguably fleeting and superseded by historically influential actors re-asserting control over data extraction and political use of digital media. Neither citizen- nor strategist-led practices are politically neutral, yet they produce different diagrams of political action through digital media. By bringing together journalistic accounts and social media coverage alongside current scholarship on political scandal and visibility, this case study traces the possibility of political accountability and the political weaponisation of user-generated content through the targeted extraction of candidate details from dispersed profiles, communities and databases. In doing so, it considers whether scandal mining is becoming a standard form of visibility for anyone seeking public office (and by extension, an emerging form of visibility in public).

## Defining political scandal

Earlier literature on political scandal provides indication of how personal incidents (of varying degrees of severity) are expressed in news media. Thompson (2000: 13–14) identifies five features of scandal: (1) transgressions of certain norms or values that (2) were indented to remain concealed, but become known to others, (3) who disapprove of these transgressions, (4) publicly express this disapproval and (5) may damage the reputation of those involved. First, the transgression itself may be categorised in terms of ‘financial corruption, political corruption, personal scandals, and international scandals’ (Basinger and Rottinghaus, 2012: 218). We may anticipate that the opening up of candidates’ lives via social platforms affords a preponderance for personal scandal, if only because one struggles to imagine evidence of financial and political corruption manifest through a candidate’s public posts. As well, we may consider ‘talk scandals’ as an additional category of speech-acts that transgress discursive norms about ‘how one should behave in the public sphere’ (Ekström and Johansson, 2008: 64). Not only can such utterances be situated within a social media platform at their conception, but they can easily circulate through a broader digital media landscape. Examples include transgressive public statements, but also instances where ‘back-stage utterances’ are rendered public. Speech-acts contained on (and circulated through) social media platforms further complicate distinctions between front- and back-stage (Trottier, 2013; Goffman, 1959), as statements presumed to be fit for circulation in the interpretation of a context-specific public (such as the fellow members of a regional or hobby-based social media group) may still provoke offence and recourse if they circulate to a broader public (such as through coverage by a national broadcaster or newspaper).

Second, the process concealment and discovery are ‘often characterised by a *drama of concealment and disclosure*’ (Thompson, 2000: 18, emphasis in original). On first pass, this drama may be greatly diminished in the case of digital media scandals, when offending acts are posted on public profiles. Yet the retroactive management of online content (deleting posts; augmenting privacy settings) may nevertheless constitute a ‘second order transgression’ (Thompson, 2000: 17), which can potentially overshadow the original offence. Third, discovery without disapproval by some kind of audience will not constitute a scandal. In considering the role of digital media, revelation and disapproval followed by silence (or online chatter that fails to reach an indeterminate threshold) may not suffice either. Scandal is partly enacted by ‘opprobrious discourse’ (Thompson, 2000: 20), including condemnation, reproach and rebukes. Thompson comments that the intensity and perhaps also the veracity of such morality-based discourse are lessened in contemporary media culture, and may instead serve partisan or policy ends. Nevertheless, the response must be uttered ‘in a way that it can be heard by a plurality of others’ (Thompson, 2000: 21) in order to produce ‘consistent views and widespread anger among the audience’ (Kepplinger et al., 2012: 659). With digital media tools ‘individuals can express opprobrium in ways which, by virtue of the medium itself, endow the expressions with the status of public speech-acts’ (Kepplinger et al., 2012). Yet, we can consider the amount of circulation or plurality necessary for such speech-acts to trigger scandal. If a social media–based talk scandal in the 2015 election was discovered and circulated by a blog with a few thousand readers, but not re-circulated by the national broadcaster or major newspapers, can it be considered as scandalous? Finally, damaged or depleted reputation is neither necessary nor inevitable, but the risk of it is. While this has been the end-state for many 20th century scandals, the magnitude of this depletion (for the individual candidate as well as the political party and its leader) may be lessened depending on the status of the political actor targeted by scandal mining. Beyond individual or party reputation, we can consider scandal’s broader impact on ‘the perceived boundaries of public institutions, either reinforcing or blurring traditional lines of demarcation between the political class, the media, the judiciary and corporate interests’ (Fieschi and Heywood, 2004: 290).

## Mediated visibility as constructed and contested

In explaining a growing prevalence of political scandal, Thompson (2000: 108) provides three attributions. First, an increased visibility of political actors produces vulnerabilities for candidates. Media publicity is a requirement for political life, while simultaneously binding aspiring politicians to other actors and unanticipated and unmanageable circuits of information exchange. Second, changes in technologies of communication and surveillance such that supposedly private conversations ‘may unexpectedly acquire a public character’ (Thompson, 2000: 109). These features are addressed in further detail below. Third, we can consider a changing culture of investigative journalism. To this we may specifically consider actors that are placed at the margins of conventional journalism, such as online semi-professionalised or crowdsourced venues. This is considered in the following section.

Visibility stands as a central condition (Lyon, 2002) and category (Brighenti, 2007) for the social sciences. Recognising this importance implies a need to appreciate the deliberate effort taken to produce and acknowledge social visibility. In the context of political scandal, it is not simply a matter of discrediting details being revealed (or leaked). In addition to discrediting content, scandal depends on the media and the public, who must accept the event as both having occurred and being offensive. In pointing to perceived shortcomings in the literature, Welch (2007) addresses an overemphasis on the determinant nature of the moment of exposure, at the expense of understanding ‘the prolonged and contested process through which that exposure occurs and is made significant’ (p. 182). Focusing on exemplary 20th-century America cases such as Watergate and the Clinton–Lewinsky affair, he advocates for an anti-essentialist understanding of political scandal ‘characterized not by exposure but by the political construction of exposure’ (Welch, 2007: 187). In contrast to the epistemologically simplistic ‘smoking gun’ metaphor, the notion of plausible deniability ‘expresses its politicized complexity’ (Welch, 2007: 188). While the construction and management of scandal warrants greater scrutiny, we may presume that in the case of lesser political figures (including inexperienced candidates in unfavourable ridings), established parties prefer the strategy of removing disgraced candidates, rather than denying the scandalous nature of social media postings. Yet treating mediated visibility as ‘always shaped by a broader set of cultural assumptions and frameworks’ (Thompson, 2005: 36) supports an understanding of scandal that is necessarily co-construction by political, journalistic and other mediated actors and contexts. The recent focus on social media gaffes further complicates this co-construction, as platforms where the offending act is authored and circulated are prominently used in a range of personal and professional contexts.

Indeed, social media are often framed as indispensible tools for politicians in elections, notably in order to reach and mobilise younger voters (Aldrich et al., 2016). Yet recent scholarship also suggests that politicians generally make use of platforms like Facebook and Twitter for ‘the more familiar and conventional logic of [a] one-way flow’ of party-approved content (Ross et al., 2015: 266), and that those who actively make use of these platforms tend to hold a kind of political ‘underdog’ status as they are ‘younger, in opposition and out of the political limelight’ (Larsson and Kalsnes, 2014: 653). This suggests a generalised reluctance among established political actors to go beyond a conservative and calculated presence on these platforms. Under these circumstances, scandalous speech-acts may play a greater role in shaping public understandings of political engagements online. Not only do citizens come to expect and seek out available content linked to politicians, but scandalous revelations may be found on accounts also used for strategic ends by candidates, notably those bearing an ‘underdog’ or ‘nobody’ status.

The openly available and easily re-circulated nature of social media content appears to dampen the possibility of political actors engaging in denial and/or concealment of scandalising content. In providing a more rigorous account of candidate reactions to these discoveries, it is possible that they invoke privacy and/or unsanctioned visibility in defending their reputation (and delegitimizing the revelations). As such, these revelations and the kinds of appeals to privacy that are (not) made speak to the negotiated status of such personal or political data. And while citizens may be in a position to locate and remediate incriminating content from political actors, these conditions of visibility may also shape how their own online visibilities are understood and negotiated.

## A shifting news media landscape?

The press are typically understood as fulfilling multiple roles in the construction of political scandal. These include setting ‘a stage for the denouncer to suggest scandal’, further legitimating the proposed scandal by ‘react[ing] to make the pattern evolve from the stage of suggesting scandal to a fully developed one’ and finally serving ‘as a proxy – or more formally speaking, as a functional equivalent – for the public in scandal communication’ (Esser and Hartung, 2004: 1047–1048). Formal media outlets are thus understood as not only providing the conditions for an initial revelation, but also for the subsequent denouncement and deliberation. Such an emphasis on scandal may serve to trivialise popular understandings of political process (Fieschi and Heywood, 2004: 299), although politicians themselves may exploit ‘the media’s shift to a more personalised content’ as a means ‘to bypass more conventional party-based channels of communication with the electorate’ (p. 300).

Whereas potentially scandalous information was previously withheld and brokered by specific gatekeepers, Williams and Delli Carpini (2004) describe the contemporary digital media landscape as ‘providing virtually unlimited sources of political information (although these sources do not provide anything like an unlimited number of perspectives)’ that ‘undermines the idea that there are discrete gates through which political information passes: If there are no gates, there can be no gatekeepers’. (p. 1208). Digital media tools can be broadly understood as enabling greater citizen engagement, notably in the manifestation of citizen journalism as well as user-led campaigns (Tufekci and Wilson, 2012). They also allow citizens to identify and circulate potentially discrediting information about political actors. Indeed, recent scholarship considers how news media practices can adapt to user-led digital media activity. Chadwick (2011) refers to the emergence of ‘nonelite participants’ who ‘now interact exclusively online in order to advance or contest specific news frames or even entire stories’ (p. 8). The fact that social media platforms in particular are both a source of scandalous data as well as spaces to further publicise this content supports the view that coverage of scandal ‘takes place in public or semipublic online environments’ (Chadwick, 2011). Yet empirical studies suggest that ‘viral’ spikes in social media viewership of a scandalous event depend on coverage from mass media venues (Toepfl, 2011). News may ‘break first online’ and then be picked up, circulated and even validated by the press (Chadwick, 2011: 5). The degree to which political events are rendered meaningful and visible nevertheless continues to depend on press organisations.

The role that digital media might play in the propagation of scandal needs to be understood more in terms of how these platforms ‘function in tandem with other spheres of traditional mass media rather than as isolated forms of communication’ (Chadwick, 2011: 1315). It is necessary to examine the interactions between digital media and other media spheres as co-constitutive of scandal. Individuals engaged in scandal mining may depend on conventional media channels in order to reach a sufficient audience, but also for the designation of a mediated act as scandalous. For this reason, cases that are reported online and not picked up by broadsheets and major broadcasters are especially illustrative of this mutual co-construction of scandal. Yet even if the role of the citizen journalist in reporting scandal is limited and dependent upon more established media channels, this marks a departure from previous understandings of the public as the mere recipient of the scandalous event (especially if mass media served as a more effective proxy for the public than members of the public themselves). This fits an understanding of citizens as an audience ‘who have had their fill of scandalous disclosures’ (Thompson, 2000: 88), contributing both to a weariness towards the media, as well as a generalised distrust of politics. While digital media may offer a novel engagement to mitigate these effects, they are also characterised by a so-called engagement economy (McGonigal, 2008) whereby user input is itself a scarce and fleeting resource. Potentially engaging through social media platforms enables citizens to mobilise fellow citizens, for example, in the context of discrediting a political candidate. Yet this potential does not obviate the need for scandal to flow through conventional media channels.

## Scandal mining in the context of the 2015 Canadian federal election

The 2015 Canadian federal election was first announced on Sunday, 2nd of August. With the election date set for the 19th of October, the 78-day campaign period would be the longest in recent history, and longer than the previous two federal elections combined.^4^ The incumbent Conservative Party, led by Stephen Harper, had been in power for the last 9 years and held a parliamentary majority since 2011. Among the other main parties, the Liberals and Bloc Québécois had each suffered historic setbacks in the previous election, with both party leaders failing to secure their own ridings. The left-leaning New Democratic Party (NDP), having made significant gains as official opposition, had suffered setbacks of their own with the sudden passing of former leader Jack Layton. Since 2011, Prime Minister Harper had lost several of his key ministers and other party-faithful members and was also considered to be in a relatively precarious position. These conditions reinforced a socio-political climate where the four biggest federal parties faced a costly electoral period, with little reassurance of any easy gains.

Already prior to the election call, three federal candidates were identified as a result of political missteps. Béatrice Zako from the NDP resigned in June after being identified as favouring Quebec independence on another (provincial) party website,^5^ while the Liberal’s Ray Fox and the Conservative party’s Julian DiBattista had published offensive content of their Facebook profile and blog, respectively.^6,7^ In the week following the election call, three candidates from the Liberal and Conservative parties, along with political analyst Jean Lapierre, were targeted by Montreal-based newspapers *La Presse* and the *Journal de Montreal*. In the following weeks, unaffiliated blogger Robert Jago and political content start-up True North Times (TNT) were collectively responsible for the discovery and reporting of at least 15 cases on Facebook and Twitter, but also YouTube, Tumblr as well as personal blogs and comments posted on news websites. These actors stood alongside more conventional news media such as the national broadcaster (CBC), prominent newspapers such as the *Globe* and *Mail* and the *Toronto Star*, as well as online news venues like Huffington Post Canada. While the latter did first identify some candidate missteps, such as Conservative candidate Jerry Bance’s infamous urination into a client’s coffee cup,^8^ they also provided Jago and TNT greater circulation by reporting their discoveries.

During the 11-week campaign, a constellation of actors, including unaffiliated or covertly affiliated individuals and organisations as well as journalists and political professionals rendered candidate missteps visible. The line separating journalistic and independent actors from major party campaigns is not evident. The website MeetTheNDP.ca took aim at the official opposition, and circulated potentially controversial perspectives expressed online. While nominally similar to sites like Conservatives of FB,^9^ a Facebook page that targets and circulates the views of semi-anonymised Facebook users, MeetTheNDP was in fact registered to the Conservative party.^10^ Such sites indicate that scandal mining marks a convergence of established party attack ad strategies and broader online campaigns of public shaming through re-circulation.

In terms of alleged offences, candidates like the Bloc Québécois’ Chantal-St-Onge had expressed support for extremist groups like Pegida,^11^ while others like the Conservative’s Blair Dale and Gordon Giesbrecht expressed controversial views about abortion.^12,13^ Interestingly, the TNT identified Liberal Kimberley Love’s anti-gun rhetoric as potentially incompatible with her rural Ontario constituents,^14^ and NDP candidate Ethan Rabidoux’s pro-gun sentiment as incompatible with his party.^15^ Thus, the expression of scandal seems based on any available political incompatibility, rather than from a personally rooted sense of moral offence. Yet the above examples of political incompatibility were overshadowed by cases of sexist, racist, xenophobic and generally vitriolic sentiment, which made up a significant part of the documented offences. Of the 39 candidates who were targeted online, 18 resigned or were removed by their party (along with Conservative board member Sue MacDonell), while the other half apologised, transferred blame to other members of staff or simply did not acknowledge the revelation. When looking at the candidates who were targeted, only three (Buddy Ford, Nicola Di Iorio and Jerry Bance) did not involve content those candidates previously posted on social media, comments sections in online news sources or other websites. Social media features predominantly in the public expression of political scandal, as for both party-affiliated and autonomous actors it is not only a platform to express and announce the scandals but also the platform where the gaffe occurs. As Facebook and other platforms become sites for user-led production and circulation of content, these actors begin to maintain assumptions about candidates: that they have some kind of public presence on social platforms (especially as political hopefuls) and that this presence contains incriminating statements.

Of the 39 targeted candidates, only one was an incumbent: NDP Pat Martin, whose offending utterances occurred at a public forum.^16^ Likewise, only 3 of 39 candidates were elected. As social media allow for a greater focus on non-political life of political figures (including those at the margins), unelected candidates become more visible and legible in election media. As it becomes taken for granted that all candidates are present on digital media (for electoral as well as in other professional and personal capacities), marginal political party members may have a greater bearing on the success of their parties. Candidates identified in scandal mining often were based in ridings where the party had little historic evidence of former success, such as Liberals in Calgary and Conservatives in Toronto. These non-incumbents could be assumed to be comparatively inexperienced in public political communications, and for strategic reasons not as carefully vetted by their party. Yet the previous election also demonstrated that the predictability of these ridings was not assured, particularly as when the NDP’s electoral gains in Quebec placed a party-affiliated type of ‘political nobody’ into the public eye.

The content used to scandalise political actors in the 2015 elections dates as far back as 2005. While political strategists may look back at 2015 as a formative moment in social media liabilities, social media platforms, blogs and comments sections on news websites were by no means a novelty at that point. Political parties and prominent candidates employed digital media strategists. And while lesser candidates surely have forward-looking strategies for social media that are to some degree directed centrally by party strategy, they may still have to cope with online content they posted previously. We can presume that many candidates proactively remove content, but these strategies may fail to evade automated caches and vigilant (manual) screencapping, often by unaffiliated individuals and self-styled ‘political nobodies’.

We may consider the origins and motivations of this particular category of political nobody. Although unaffiliated with influential media outlets (or political parties), they use digital media to discover and announce scandal, while relying on the national broadcaster and major newspapers’ reporting and confirmation of these scandals when their discoveries are picked up on these venues. Robert Jago is a Montreal-based blogger who describes himself on Twitter as ‘[h]ead of education programs mgmt firm in Canada/US/France’.^17^ Although formally unaligned in 2015, Jago had previously volunteered for the NDP at the age of 19 and for the Conservative party in the three previous elections.^2^ At that point, he was also affiliated with the politically conservative news magazine *Western Standard* and targeted Liberal and Green candidates. He cites several reasons – including electoral reform, the fair elections act and anti-terrorism measures – as justification to target a particular type of Conservative candidate: one with little public visibility standing in tactically important ridings. In a mid-September interview with *Maclean’s* magazine, Jago refers to the next phases of his campaign, including assembling a team of writers.^2^ Jago’s YouTube channel is affiliated with a Xeylex.com, a website that ‘focuses on Canadian politics from the perspective of First Nations and people of colour’.^18^ This site is also described as

a place where our writers can talk about national politics and talk to national leaders, and where we make our stories, everyone’s stories. We are best known for our MeetTheHarperGang.com project, which saw the candidates dismissed from the Conservative Party, a half dozen more forced to apologize, and more than 200 stories that made national press, with an impact on shaping the narrative on 20 out of the election’s 78 days.^18^

It is notable that both Xeylex and MeetTheHarperGang are defunct sites as of April 2016. This point is considered below.

TNT is a website managed by a group that is partly inspired by Jago’s efforts,^19^ and yet employs more of a start-up framework in expressing its purpose and structure. They claim to produce media content ‘designed to engage the demographics that have a huge potential impact but are plagued by apathy’.^20^ Audience apathy, rather than a morality-based engagement with political process, is the impetus for the following mission statement:

Our goal is not only to make Canadian politics accessible, it is to make it funny, to make it entertaining. The Canadian political scene has never been more amusing. Scandals, politicians who are more ridiculous than cartoon characters and a highly contested election on the horizon, where there will surely be blunders and comedic moments. The True North times exists to capitalise on those moments.^20^

Their website also expresses a three-tier organisational structure featuring 3 chief staff (each with two titles like Chief Marketing Officer and Chief Strategy Officer), 3 editorial staff and 20 contributors. Although they may stand outside of conventional political parties and mass media outlets, their political experience and organisational structure is not fully captured in the term ‘political nobody’. Likewise, Liberal candidate Ala Buzreba’s offensive tweets were discovered by Sheila Gunn Reid, currently the ‘Alberta Bureau Chief’ of TheRebel, an online platform run by former Sun News Network host Ezra Levant. Online platforms like Press Progress also contribute to scandal mining, notably through the identification of Conservative candidate Marilyn Gladu’s anti-Muslim rhetoric on Facebook.^21^ Yet this particular outlet has explicit links to the progressive think-tank ‘the Broadbent Institute’ (founded by former NDP leader Ed Broadbent), and as such does not share the same kind of non-professionalised designation. Other cases may be linked to less prominent actors who remain unnamed in press coverage. Low-level scandal can also be triggered by real-time activity on digital media, for example, when Chantal St-Onge expressed support for Pegida on Facebook nearly 7 weeks into the campaign. In this case, the scandal is framed as being brought about in direct response to the candidate’s online act, and the process of seeking offensive content is greatly downplayed.

In exploring mass media coverage, there appears to be a lack of consensus in terms of which incidents are designated as such. The CBC,^22^ the *Toronto Star*^23^ and the *Globe* and *Mail*^24^ have each compiled and published lists of candidate gaffes, in addition to prior coverage given to individual cases. While there is some agreement on cases, notably those occurring in the first 2 weeks of September, beyond this point, these three compilations provide conflicting accounts of which political careers have been tarnished. Of particular note is that while the *Toronto Star* refers to ‘Social Media Gaffes’ in its compilation, both the CBC and *Globe* and *Mail* refer to gaffes in a more generic sense (with both including Jerry Bance, who was first identified as urinating in a coffee cup on national television, not on digital media). This indicates that among news media sources, there is no shared framing of 2015 electoral scandals as exclusively manifest on social media. Beyond this, the production of official lists is itself a curious phenomenon. The presence of opprobrious discourse was largely understood as a vital component of the public manifestation of political scandal; yet, in 2015, this tendency has been partly displaced by a motivation to compile and catalogue a broader array of cases involving comparatively minor political actors accused of comparatively lesser offences.

On 21 September, around the time that the aforementioned lists were being compiled, TNT announced its intention to publish discrediting online comments from nine political candidates, labelling this series ‘The Nine Days of Scandal’.^25^ While attempting to capitalise on attention given to prior cases, the nine instances of social media gaffes failed to garner the same kind of media attention,^26^ although at least two of the nine targeted candidates withdrew from the election. TNT’s campaign seems to correspond with its stated purpose to ‘capitalise’ on the mediated political landscape, notably by attempting to exert control over public talk of revelations (cf. Follis and Fish, 2017), yet it also contributes to an understanding of social media–based ‘talk scandal’ as something that can be readily extracted by individuals or organisations with sufficient tools and motivation.

While digital media platforms are thus framed as a resource that can be extracted for political and or media gains (through the visibility of missteps), the 2015 election also indicates some limitations to the kind of visibility that can be constructed. First, as discussed previously, candidates will remove incriminating content, either pre-emptively before a discovery, or shortly after it is announced or covered in the media. Such tactics mirror earlier denial and concealment moves by political actors in the grips of a scandal. What is more striking is that those involved in the extraction and circulation of scandalous content also have an ephemeral presence online. At the time of writing, neither Jago’s website (Some Random Political Blog) nor Twitter account contain any substantial evidence of the revelations made during the 2015 election campaign. Likewise, MeetTheNDP’s Twitter profile is still accessible, but all eight tweets on this account contain links to a defunct website. In these cases, the process of making scandals visible is itself no longer visible. Such findings are in line with a generalised strategic use of mediated visibility (Trottier, 2017), whereby any evidence of a mediated campaign is itself removed. They also problematise a claim by Thompson (2000) that political scandals ‘are unlikely to rely solely or heavily on relatively ephemeral forms of evidence’ (pp. 68–69). Social media content may initially be more accessible to the public than other evidence of scandal, notably through the re-circulation of offending content by investigative journalists and ‘political nobodies’. Yet if the offending content and the initial discovery can both be removed by their respective perpetrators, it bears reflecting on the ephemerality of such campaigns, with evidence of the offence and ensuing campaign accessible only through secondary press coverage and archived caches.

## Discussion

Electoral scandals in 2015 involved two sets of political nobodies: unelected candidates in unfavourable ridings and individuals who render past missteps visible through remediation. Whereas political scandals formerly centred on elected officials and the disclosure of deliberately withheld (private) information, scandal mining pre-emptively targets political hopefuls on the basis of content they posted on social media platforms with quasi-public designations. Press and other public coverage of these cases do not appear to be framed in terms of privacy violations, or about personal/professional distinctions. While many of the cases pertain to content on personal social media accounts, public scrutiny of this content is largely uncontested. While these individual disclosures ended the political aspirations of many candidates, they are also indicative of contemporary conditions of visibility online, as well as of the potential for one form of citizen-led political engagements. This exploratory case study invokes the potential that those formally unaffiliated with politics or journalism can intervene in the political process by finding compromising information about candidates and circulating this through media channels. At least in this respect, journalists, political agents, bloggers and designated ‘nobodies’ are not differentiated in terms of the skills or information access they possess. Consisting of ‘loosely coupled assemblages characterized by conflict, competition, partisanship, and mutual dependency’ (Chadwick, 2011: 19), scandal mining may potentially transcend issues of access that characterise big data science (Chan, 2015). Although dependent on a broader infrastructure that includes social media platforms, these can be accessed through more rudimentary and scaled-down entry points.

Although the affordances associated with digital media extend the possibility of political scandal, the 2015 election saw the emergence of a type of politician to be targeted: candidates from major parties with comparatively lesser profiles placed in strategic and/or unwinnable ridings. They had formal party affiliation and as such could harm its reputation, while seemingly lacking adequate professionalisation in the mediated public eye. We can speculate whether personal social media posts from up to a decade ago were authored with the potential of political and public scrutiny in mind. Likewise, the data sources that are drawn upon are also typically limited to known social media profiles as well as comments sections on prominent news sites. Subsequent research should consider scandal mining in relation to commercially available searching technologies, especially as party strategists may be the only political actors with budgets dedicated for them. And while some of the actors performing scandal mining are formally unaffiliated, they typically have many years of experience with prior forms of political engagement (in Jago’s case), or engage in such practices through a formal organisational structure (in TNT’s case). Furthermore, while scandal mining offers the possibility of targeting candidates on grounds other than partisan strategy, we see that this was not the case in Jago’s campaign (as he exclusively targeted Conservative candidates in 2015), nor was it with ‘astrosurfing’ (Boulay, 2015) campaigns such as MeetTheNDP. Here, we may at least begin to distinguish between value-based partisan engagements with data, and those based primarily on party affiliation. Consider the distinction between seeking and denouncing social media activity that contravenes one’s own political commitments (such as gun control), and attacking a political party by amplifying the visibility of a candidates’ statement on any available controversial issue such as gun control, regardless of the position itself. Subsequent research should also consider the extent to which such non-neutral forms of politics are aligned with both traditional and entrepreneurial forms of populism (Fieschi and Heywood, 2004). Comparing Jago and TNT in particular, the latter appears to mobilise scandal in order to generate visibility of both the political candidates, but perhaps more importantly their own website. The case described above marks a hybrid mobilisation movement: the social construction of scandal is manifest as fleeting multi-partisan or even non-partisan assemblages of media and political actors, who coalesce in an electoral context despite conflicting political and entrepreneurial goals. Any of these goals appear to depend on circulation through mainstream news outlets, which in turn make use of this content to feed daily news cycles.

On these grounds, we may instead consider the manner in which scandal mining may further existing asymmetries in political processes, as well as facilitate pre-existing and predominant forms of management and control through digital media. While the four major political parties (along with major broadcasters and broadsheets) might not have anticipated the extent to which candidate missteps were brought into the public eye, it is fair to presume that these events will compel them to take a more rigorous and proactive approach to candidate screening, or cyber-vetting (Berkelaar and Buzzanell, 2014). Such developments, notably in the anticipation of low-level political scandals, would further a paradox of mediated political processes: scandals are by definition an upset to politics, yet an increased frequency (and decreased severity) of such indiscretions facilitates a series of pre-emptive strategies, purported best practices and shared framing of scandal among conventional political and journalist actors. Further research can consider how news media as well as users express such discoveries on digital platforms, notably through concepts such as scandal and gaffe, but also offence, incrimination and discrediting. In considering offence-taking as a key juncture in scandal as a process, the fact that incidents were not identified based on internally consistent moral values suggests that we may not only be moving towards post-ideological scandal (Meng, 2016) but even towards a post-offence practice where any available narrative will be invoked in order to harm the public standing of a targeted individual or political party.

Citizens are able to handle unanticipated and scandalising data; yet, the 2015 case suggests that they still depend on conventional media venues to actualise the moment of exposure, and thus effect some form of political action. Moreover, the extent to which scandal mining can be utilised as a form of public accountability seems limited if it predominantly targets low-level politicians, without putting major parties (or more influential political actors) under increased scrutiny. As such, this form of accountability is not dissimilar to the scrutiny that a broader category of (job) candidate may experience, as opposed to rendering politics visible in a more substantive or systemic manner. Jago himself laments the fact that the media have given greater attention to the relatively trivial social media gaffes, as opposed to his attempts to uncover anti-aboriginal discrimination.^27^ While citizen-led engagements may have limited impacts on public understandings of politics, one possibility for public accountability might lie in attempts to render party-funded scandal mining campaigns visible.

## Notes

1. www.ontariosunshinelist.com (accessed 16 October 2017).

2. http://www.macleans.ca/politics/ottawa/robert-jago-the-most-dangerous-blogger-in-canada/ (accessed 16 October 2017).

3. http://www.cbc.ca/news/politics/canada-election-2015-vetting-candidates-media-1.3229594 (accessed 16 October 2017).

4. http://ottawacitizen.com/news/politics/two-down-76-to-go-the-longest-election-campaign-since-we-first-re-elected-john-a (accessed 16 October 2017).

5. http://www.cbc.ca/news/canada/montreal/b%C3%A9atrice-zako-resigns-as-ndp-candidate-after-sovereigntist-revelations-1.3159691 (accessed 16 October 2017).

6. http://www.huffingtonpost.ca/2015/06/19/ray-fox-liberals-facebook_n_7620260.html (accessed 16 October 2017).(accessed 16 October 2017).

7. http://www.metronews.ca/news/toronto/2015/08/06/conservative-toronto-candidate-apologizes-for-comments.html (accessed 16 October 2017).

8. http://www.cbc.ca/news/politics/jerry-bance-marketplace-1.3217797 (accessed 16 October 2017).

9. https://www.facebook.com/conservativesss (accessed 16 October 2017).

10. http://www.thestar.com/opinion/commentary/2015/08/17/when-attack-ads-backfire.html (accessed 16 October 2017).

11. http://montrealgazette.com/news/quebec/bloc-quebecois-candidate-says-she-is-profoundly-sorry-for-facebook-post (accessed 16 October 2017).

12. http://www.cbc.ca/news/politics/canada-election-2015-blair-dale-newfoundland-cpc-1.3229260 (accessed 16 October 2017).

13. http://www.cbc.ca/news/canada/manitoba/gordon-giesbrecht-tory-candidate-9-11-abortion-video-1.3239298 (accessed 16 October 2017).

14. http://www.truenorthtimes.ca/2015/09/27/liberal-candidate-kimberley-love-compared-alberta-oil-sands-mordor/ (accessed 16 October 2017).

15. http://www.truenorthtimes.ca/2015/09/28/ndp-rabidoux-gun-democracy/ (accessed 16 October 2017).

16. http://www.cbc.ca/news/canada/manitoba/candidate-wants-apology-ndp-to-dump-pat-martin-for-son-of-a-bitch-comment-1.3231387 (accessed 16 October 2017).

17. https://twitter.com/rjjago (accessed 18 April 2016).

18. https://www.youtube.com/channel/UCM0kokV51eUpexYWzGkVoyA (accessed 18 April 2016).

19. http://www.truenorthtimes.ca/2015/10/19/true-north-times-endorsement-democracy/ (accessed 16 October 2017).

20. http://www.truenorthtimes.ca/about/ (accessed 16 October 2017).

21. http://www.pressprogress.ca/this_conservative_candidate_is_not_happy_about_the_separation_church_and_state (accessed 16 October 2017).

22. http://www.cbc.ca/news/politics/canada-election-2015-candidates-dropped-gaffes-in-trouble-1.3231366 (published 16 September, last updated 1 October, accessed 16 October 2017).

23. http://www.thestar.com/news/federal-election/2015/09/23/social-media-bites-yet-more-election-candidates.html (published 23 September, accessed 16 October 2017).

24. http://www.theglobeandmail.com/news/politics/full-list-of-candidates-party-officials-who-have-made-election-gaffes/article26415839/ (published 17 September, last updated 28 September, accessed 16 October 2017).

25. http://www.truenorthtimes.ca/2015/09/21/candidate-scandals-election-exclusive/ (accessed 16 October 2017).

26. http://ipolitics.ca/2015/09/29/the-true-north-times-wraps-up-candidate-scandals-series/ (accessed 16 October 2017).

27. https://www.buzzfeed.com/ishmaeldaro/blog-against-the-machine (accessed 16 October 2017).
